# Hyperspectral and Radar Airborne Imagery over Controlled Release of Oil at Sea

**DOI:** 10.3390/s17081772

**Published:** 2017-08-02

**Authors:** Sébastien Angelliaume, Xavier Ceamanos, Françoise Viallefont-Robinet, Rémi Baqué, Philippe Déliot, Véronique Miegebielle

**Affiliations:** 1 Electromagnetism and Radar Department, ONERA, BA 701, 13661 Salon Air, France; Remi.Baque@onera.fr; 2 Optics and Associated Techniques Department, ONERA, 2 avenue Edouard Belin, 31055 Toulouse, France; Xavier.Ceamanos@onera.fr (X.C.); francoise.viallefont@onera.fr (F.V.-R.); Philippe.Deliot@onera.fr (P.D.); 3 Research and Development Department, TOTAL, avenue Larribau, 64000 Pau, France; Veronique.Miegebielle@total.com

**Keywords:** SAR imagery, hyperspectral imagery, radar sensor, optical sensor, multi-frequency, multi-wavelength, oil slick, oil spill, seeps, maritime pollution

## Abstract

Remote sensing techniques are commonly used by Oil and Gas companies to monitor hydrocarbon on the ocean surface. The interest lies not only in exploration but also in the monitoring of the maritime environment. Occurrence of natural seeps on the sea surface is a key indicator of the presence of mature source rock in the subsurface. These natural seeps, as well as the oil slicks, are commonly detected using radar sensors but the addition of optical imagery can deliver extra information such as thickness and composition of the detected oil, which is critical for both exploration purposes and efficient cleanup operations. Today, state-of-the-art approaches combine multiple data collected by optical and radar sensors embedded on-board different airborne and spaceborne platforms, to ensure wide spatial coverage and high frequency revisit time. Multi-wavelength imaging system may create a breakthrough in remote sensing applications, but it requires adapted processing techniques that need to be developed. To explore performances offered by multi-wavelength radar and optical sensors for oil slick monitoring, remote sensing data have been collected by SETHI (Système Expérimental de Télédection Hyperfréquence Imageur), the airborne system developed by ONERA (the French Aerospace Lab), during an oil spill cleanup exercise carried out in 2015 in the North Sea, Europe. The uniqueness of this dataset lies in its high spatial resolution, low noise level and quasi-simultaneous acquisitions of different part of the EM spectrum. Specific processing techniques have been developed to extract meaningful information associated with oil-covered sea surface. Analysis of this unique and rich dataset demonstrates that remote sensing imagery, collected in both optical and microwave domains, allows estimating slick surface properties such as the age of the emulsion released at sea, the spatial abundance of oil and the relative concentration of hydrocarbons remaining on the sea surface.

## 1. Introduction

In the context of offshore hydrocarbon exploration and production, Oil and Gas (O&G) companies thrive to discover new oil fields. It is also their duty to exploit them in a clean and safe manner. Field exploration demands more and more efficient and high performance techniques to discover new reservoirs in increasingly extreme environments. This exploration area has a high probability of being located under a large body of water that does not allow direct access to the bottom of the ocean and requires the use of long range interpretation techniques. Early detection of the occurrence of natural seeps, on the ocean surface, is a key indicator of the presence of mature source rock in the subsurface. Furthermore, remote sensing technology [1,2] is also essential in the global monitoring of offshore production sites. The ability to respond rapidly, based on accurate information, to threatening incidents enables O&G companies to minimize the impact and to reduce risk of offshore operations [3].

Today, optical and radar sensors are currently available either from space or onboard aircraft. Combining multi-wavelength imagery appears like a breakthrough in remote sensing applications [4,5], but it requires specific processing techniques. Methodologies for detecting hydrocarbons at sea are typically based on radar imagery [6,7], mainly as a result of weather-related issues that require data on which cloud cover has little impact.

Oceanic and atmospheric phenomena (called look-alike) can occur over the ocean surface and manifest themselves on radar images in the same way (dark patches) as area covered by hydrocarbons. Distinction between natural oil seeps, oil spill (accidental event) and radar look-alikes, such as phytoplankton bloom or low wind areas, may be tricky when looking only at radar data, but could be done by combining radar and optical remote sensing data. Optical and radar imagery collected over the same area and almost in the same time, allows scientists to progress in this issue. In addition, the complementarities of multi-frequency radar data [8] and multi-wavelength optical data [9] are also an important way of improvement in the context of oil-slick monitoring.

To assess and solve these issues, TOTAL (the French petroleum company) and ONERA (the French Aerospace Lab) have for several years embarked upon the development of innovative remote sensing techniques to detect and monitor ocean surface covered by hydrocarbons, having in mind both the exploration and the environmental monitoring of off-shore operations. The activities, which are part of the NAOMI (New Advanced Observation Method Integration) project, investigate performances in the field of detection, characterization and quantification of slick-covered sea surface provided by radar and optical imagery.

To sustain these activities, remote sensing data have been collected by SETHI (Système Expérimental de Télédection Hyperfréquence Imageur), the airborne system developed by ONERA, over controlled release of mineral oil at sea during the oil-on-water (OOW) exercise carried out by NOFO (Norwegian Clean Seas Association for Operating Companies) in 2015 off the Norwegian coast. This experiment at sea is primarily dedicated to testing mechanical recovery systems. Several sensors were operated onboard SETHI and have imaged the spill areas: dual-frequency Synthetic Aperture Radar (SAR) imaging in a full polarimetric mode and optronic sensors composed of one panchromatic camera (aligned with the radar viewing angle) and two hyperspectral cameras (nadir imagery) covering the full range of 0.4–2.5 µm with a very high spectral resolution.

This paper presents the airborne sensors operated by ONERA during the oil spill cleanup exercise organized by NOFO in 2015 and demonstrates the added-value brought by multi-wavelength remote sensing imagery collected in both optical and microwave domains to carry out oil-slick monitoring. It is organized as follows: Section 2 describes the experiment at sea managed by NOFO, Section 3 presents in detail the remote sensing sensors operated onboard SETHI during the airborne campaign of measurements, Section 4 gives the results obtained in both optical and microwave domains and main discussions are in Section 5.

## 2. Experimentation at Sea

During the oil spill cleanup exercise organized by NOFO in 2015, mineral oil was released at sea in a control manner, with the main objective of testing mechanical recovering system in a realistic marine environment. The oil-on-water exercise was carried out in the Frigg field (located in the North Sea between Norway and the United Kingdom, 230 km northwest of Stavanger) within 10 Nautical Miles of position (59°59′ N, 02°27′ E), from 8 to 14 June.

**Figure 1:**
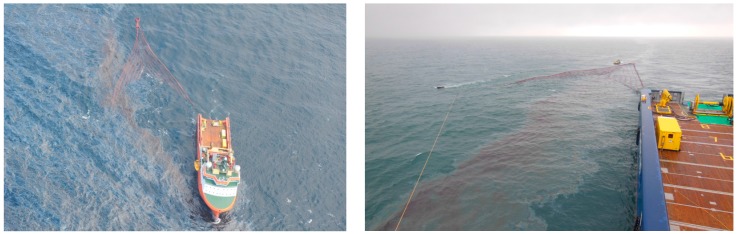
MOS Sweeper [10] mechanical recovering system operated by NOFO during trial at sea (photographs provided by NOFO).

In this paper, we focus on remote sensing data acquired on 9 June, during the so-called MOS Sweeper exercise (Figure 1). MOS Sweeper [10] is a multi-barrier system in V-shaped pattern operated from a single vessel, which guides the oil towards the rear and center of the system where an integrated pump transports the oil directly to the towing vessel. For the experiment, hydrocarbons are released from a floating pump at the sea surface and towed by another vessel (Stril Mariner). The distance between the two vessels is only a few hundred meters.

MOS Sweeper trial began on 9 June in the early morning (06:00 a.m. UTC) and continued until 10:30 a.m. UTC. After a first phase of low flow discharge (06:00–06:30 a.m. UTC), the release continued between 06:30 a.m. and 08:00 a.m. at full regime. The boats were advancing towards the west-northwest (heading 290°) at a speed of 2.5 knots. Around 08:00 a.m., the lead vessel (Stril Mariner) had completed the release and Stril Luna, the boat towing the MOS Sweeper system, turned around and continued the cleaning process of the slick until 10:30 a.m. At the time of data acquisition, it was heading towards the east-southeast (heading 100°) at a speed of 2.5 knots, following the drift of the oil slick.

The released product is an emulsion of mineral oil in water, which was produced by NOFO and consists of a mix of water, Oseberg crude oil and a small addition of IFO 380 (Intermediate Fuel Oil or marine diesel oil, with viscosity of 380 mm^2^·s^−1^). The water content of the recovered emulsion was measured by NOFO and is equal to 60%. For the trial, 45 m^3^ of mineral oil emulsion was discharged at sea and recovered by the MOS Sweeper system.

During the experimentation, sea state information was provided by the Norwegian Meteorological Institute and is given in Table 1. This information is consistent with that measured from one of the vessels present during the offshore experiment and given in [11].

## 3. Airborne Campaign of Acquisition

SETHI is the ONERA airborne remote sensing laboratory [12] designed to explore the science applications of remote sensing. It is onboard a Dassault Aviation Falcon 20 that can fly up to 30,000 ft above sea level, with 2.5 h of autonomy.

**Figure 2:**
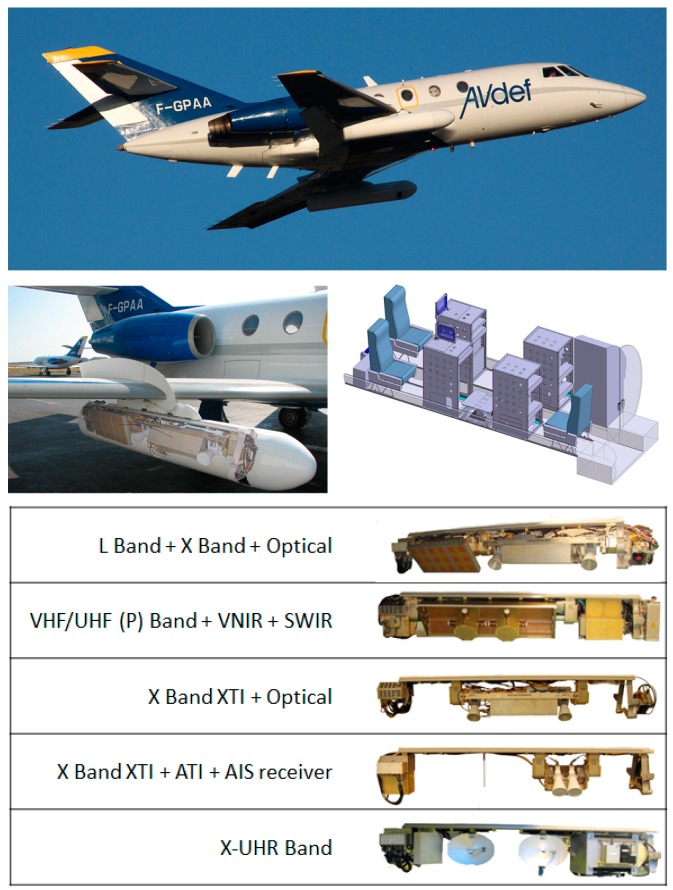
SETHI platform (aircraft, pod and cabin layout) and in-pod payload configuration examples.

**Figure 3:**
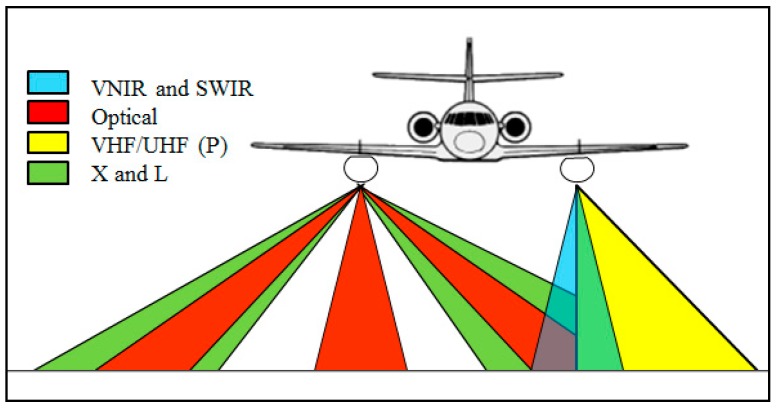
SETHI—possible geometries of imagery for both optical and radar sensors.

It combines two pods under wings which are able to carry heavy and cumbersome payloads (2.3 m long, 53 cm diameter and 120 kg of payload each pod) ranging from VHF-UHF to X-band and optical sensors with a wide range of acquisition geometries as shown Figure 2 and Figure 3. The optronic sensors offer very high spatial resolution visible images (0.4–0.8 µm) and fine spectral scene analysis in the VNIR (0.4–1.0 µm) and SWIR (1–2.5 µm) ranges. The pod-based concept allows the easy integration and testing of new systems under the single certification of the pods by authorities. Radar payload configuration flexibility allows full polarimetric and very high resolution modes (up to centimeter), along track interferometry (ATI) and across track interferometry (XTI) and very high precision multi-baseline capacity for interferometry and tomography applications.

The cabin (88 U and 480 kg) and pod capacities allow payload boarding flexibility that justifies SETHI interest as an airborne remote sensing research platform. Its flexibility is also demonstrated regarding the sensor geometry and configuration possibilities. The high dynamic and high data rate performances of the digital core are useful for large area observation (large swath) and simultaneous multi-wavelength area imaging.

The radar antennas can be oriented and moved during operation. This flexibility is provided by motorized antenna that can be horn, patches array, dipoles and parabolic antennas. The incidence angle of each antenna can be changed during the flight with a high accuracy (0.1 degree). This is a significant capability enabling to explore the incidence angle effect on clutter or target signature. This motorization is also used to point different radars on a same area for simultaneous multi-frequency imagery. Figure 3 illustrates possible geometries of imagery for both optical and radar sensors.

### 3.1. Hyperspectral Sensors

The optronic sensors, installed in the SETHI pods, are composed of one panchromatic camera (CamV2 [13], developed by the French National Mapping Agency, IGN (Institut Geographique National)) and two HySpex VNIR1600 and SWIR320m-e hyperspectral cameras built by NEO (Norsk Elektro Optikk) [14]. The main parameters of optical sensors operated during the NOFO oil-on-water exercise are given Table 2. These HySpex push-broom cameras imaging synchronously offer an effective complementarity in the spectral domain: VNIR1600 covers the full range of 410–996 nm with 160 spectral bands with a spectral sampling of 3.6 nm while the SWIR320m-e based on a Mercury Cadmium Telluride—Focal Plane Array (MCT FPA) covers the extended domain of 970–2500 nm with a spectral sampling of 6 nm. They offer a total of 410 different spectral bands providing a high spectral resolution of the scene. The spectral overlap between the VNIR and SWIR cameras allows the quick assessment of the proper calibration of the instruments. The along track and across track instantaneous field of view (iFOV) are equal to 0.37 × 0.185 mrad for the VNIR camera and 0.75 × 0.75 mrad for the SWIR camera (pixels of the SWIR camera are twice larger along track and four times larger across track than the VNIR ones). With 1600 pixels for the VNIR camera and 320 pixels for the SWIR one (across track) the resulting field of view is about 17° and 14° respectively for the VNIR and SWIR sensor.

**Figure 4:**
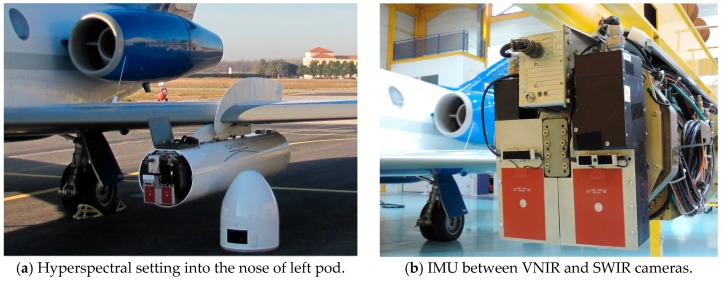
(**a**) Hyperspectral setting; and (**b**) Inertial Motion Unit (IMU).

An Inertial Motion Unit (IMU) iTrace F200 from iMAR, is located close to the two pushbroom imaging spectrographs (Figure 4b). The lever arm between cameras and IMU is small enough to ensure accurate measurements of attitude and position. Markers signal from IMU are generated synchronously with scan lines in order to georeference data in the upcoming processing.

The panchromatic camera is mounted inside the right pod. The hyperspectral ones are mounted inside the nose of left pod (Figure 4a). HySpex sensors and the IMU are fixed on the same base plate aiming approximately in the same sight angle (Figure 4b). The offset in the sight angle is processed after acquisition using ground control points. The GPS antennas are installed on the top of each pod above the camera.

To reduce time lag between radar and optical acquisitions, as explained in the next paragraph and illustrated by Figure 8, the same altitude, namely 9000 ft (Flight Level 90), driven by radar needs, is used for the two spectral domains.

To ensure a flight speed compatible with the cameras frame rates, at this altitude, the plane must fly with a ground speed around 120 m·s^−1^. This speed is quite slow for the Falcon 20, which is a jet plane, which induces a pitch angle of about 5°. This angle had to be mechanically offset to ensure a nadir line of sight. The assembly was done in France and the travel to Norway was at high altitude which implies quite low temperatures inside the pods. Moreover, to cope with temperature issue inside the pod during measurements in Norway, the optical sensors were covered by an insulated material and were maintained in a correct range of temperature with a flat heating resistance triggered by a thermostatic sensor. This heating device is also used to avoid ice deposits on the glass of the porthole.

Another issue that had to be considered concerned the vibrations inside the pod during acquisition. A specific study was carried out to measure the vibration spectrum and to eliminate its impact. First, low-frequency shock absorbers installed in the pod achieved a partial removal. The residual movement is generally at such a low frequency when it occurs that it is finally compensated by post-processing using the data from the IMU.

Dedicated software has been developed by ONERA for processing and archiving image products such as georeferenced spectral radiance and spectral ground reflectance products. Processing consisted in applying calibration coefficients on the hyperspectral images to obtain radiance products in raw geometry (level 1a), which take into account the transmission through the glass of the pod. From level 1a, image is corrected for atmospheric effects (level 2a) using the ONERA atmospheric tool COCHISE [15], which is based on the use of hyperspectral information combined with the radiative transfer code MODTRAN [16]. As scan lines are synchronized with altitude and position measured by IMU it is possible to get the position of each pixel in the image geometry map. These geo-referenced images are projected onto an UTM map grid to achieve levels 1b and 2b, i.e., ortho-rectified images in units of spectral radiance or reflectance. As the time schedule was driven by the trial of the mechanical recovering boom during the exercise at sea, it was not possible to fit the images acquisitions to the best meteorological conditions. During the NOFO campaign, most of the images were collected with the presence of sparse-cloud cover. In the following, we focus on areas that were not or slightly affected by clouds. In the latter case, the spectral reflectance obtained after atmospheric correction would be only slightly degraded.

### 3.2. Microwave Sensors

SEHTI radar sensors operated during the OOW’2015 experiment where the L- and X-band ones. The waveform used for this airborne campaign of measurements is presented Table 3.

Dual-frequency imagery is made possible by orienting both radar antennas to the same side during operation and sampling/record system high performances. The generation is performed by a 10-bit 2-channel 12 GS/s AWG (Arbitrary Waveform Generator) that transmits a pre-calculated signal. The 150 MHz bandwidth L-band signal (1.25–1.4 GHz chirp) is directly generated (no frequency up-conversion needed). The 300 MHz bandwidth X-band signal (9.6–9.9 GHz chirp) is obtained after up-conversion based on a fixed local oscillator (8.175 GHz).

The sampling system was in 10-bit 4-channel 2 GHz analog bandwidth mode. Each channel was sampled at 800 MS/s data rate and stored at 1.7 GB/s on HDD (Hard Disk Drives). These high dynamic and high data rate performances are useful for large area observation (large swath) and simultaneous multi-frequency area imaging. As for generation, L-band received signals are directly sampled (no down-conversion needed), X-band received signals are down-converted before sampling.

**Figure 5:**
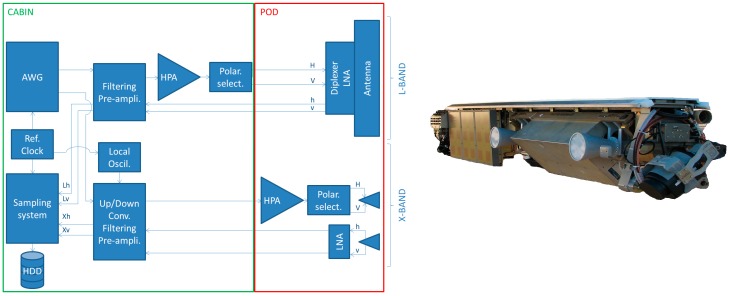
SETHI quad-polarization X+L configuration: Synoptic of sensors and view of in-pod L-band patch array and X-band horn antennas (with optical camera in the foreground).

To achieve best sensitivity and reduce the radar noise figures, LNA (Low Noise Amplifiers) have been placed inside the pod, close to the receiving antennas. To limit transmit losses, the X-band HPA (High Power Amplifier) has also been integrated inside the pod close to the transmitting antenna. Thanks to very low losses cables between cabin and pods (at low frequencies), the L-band HPA is installed inside the cabin with the AWG, the sampling/record systems and back-end microwave modules (see Figure 5).

**Figure 6:**
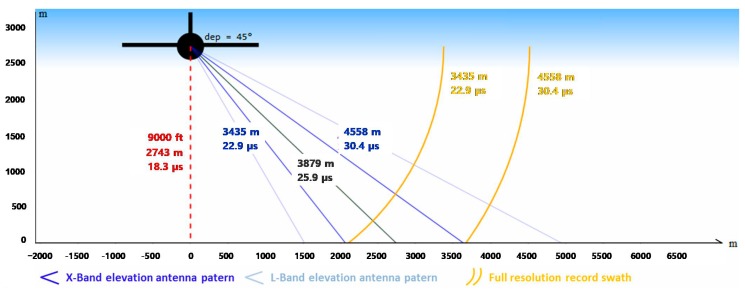
Radar acquisition geometry operated during the NOFO oil-on-water exercise.

For the OOW’2015 experiment, L- and X-band antennas are oriented at a fixed 45° of incidence angle. To reach an illuminated area of minimum 1.5 km in X-band, the minimum flying altitude is equal to 9000 ft. L-band antenna elevation aperture is bigger than X-band one so common illuminated area is limited by the X-band geometry (see Figure 6).

Radar PRF (Pulse Repetition Frequency) has been selected regarding Range and Doppler ambiguities: 10 kHz is chosen so useful PRF for each SAR polarization image is 5 kHz (each polarization—H and V—is transmitted every two radar recurrences). To maximize the radar signal to noise ratio (SNR) without reducing too much the full resolution swath (that corresponds to sampled swath duration minus pulse duration), the chosen transmitted pulse duration is 20 µs (transmit duty cycle 20%).

To record the complete illuminated area, the sampling system is configured with a 27.5 µs sampling window duration for each recurrence and each received channel. Regarding the incidence angle and the transmitted pulse duration, the obtained full resolution swath is equal to 1.57 km, in ground range geometry. This allows scene observation at incidence angles ranging from 34° to 52° across the swath. The radar acquisition trajectory is a 10 km long straight lines (90″ record duration), so SAR images cover 1.57 × 10 km^2^ areas with spatial resolution (in slant range) of 0.5 × 0.5 m^2^ in X-band and 1.0 × 1.0 m^2^ in L-band.

**Figure 7:**
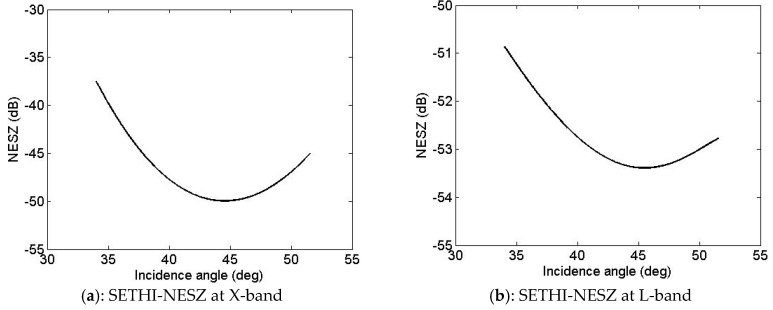
SETHI-NOFO’2015 experiment—Noise Equivalent Sigma^0^ at: (**a**) X-band and (**b**) L-band.

Waveform parameters have been selected to reach the best compromise between large swath (improved with high altitude) and high signal to noise ratio (improved with small range). The Noise Equivalent Sigma Zero (NESZ) has been estimated using the method proposed in [17]. It takes very low values (see Figure 7), ranging from around −37 to −50 dB at X-band and from around −51 to −53 dB at L-band, allowing a sufficiently high SNR for efficient analysis over sea surface covered by oil.

### 3.3. Campaign of Acquisition

**Figure 8:**
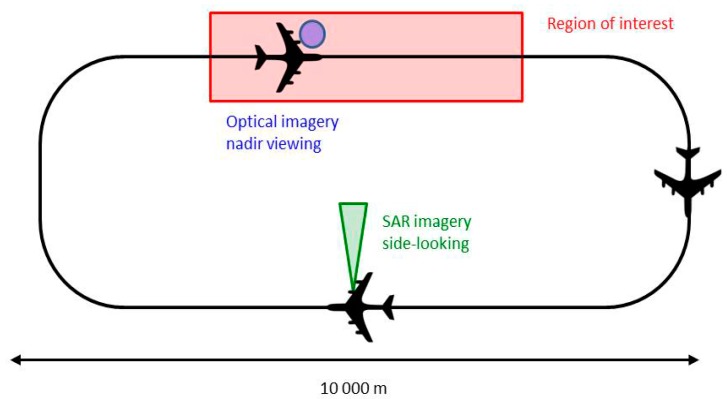
Trajectory followed by the SETHI multi-sensors during the NOFO OOW’2015 experiment.

Optical and radar sensors used for this campaign have been presented above (see Section 3.1 and Section 3.2); we focus now on the data acquisition process. SETHI was flying at an altitude of 9000 ft., along a trajectory describing a racetrack, of which longest length measures approximately 10 km (see Figure 8). The hyperspectral acquisitions (nadir viewing) are made along the first major axis of the racetrack, vertically of the oil-covered area. Side-looking SAR imageries are acquired along the return axis. Trajectories are adjusted before each run of acquisition from the AIS signal of Stril Luna (vessel towing the MOS Sweeper recovering boom and supposed to be within the slick) received in real time onboard the SETHI system. Flying at a ground speed of 120 m·s^−1^, the time lags between optical and radar imagery is about a few minutes. A description of each run of remote sensing data acquisition carried out during the MOS Sweeper trial is given Table 4. In this table, the lines in bold correspond to the acquisitions mainly analyzed in the following, namely dual-frequency SAR imagery at 10:01 a.m. UTC (run 8) and VNIR-SWIR hyperspectral imagery at 10:12 a.m. UTC (run 10).

## 4. Hyperspectral and Radar Imagery over Oil Slick

### 4.1. Imagery in Optical Domain

Discrimination between oil slicks and other phenomena (look-alike) was shown to be feasible with the use of optical imaging technique [18]. Furthermore, distinction between oil types and indications on oil volume can be achieved thanks to hyperspectral imaging [19].

**Figure 9:**
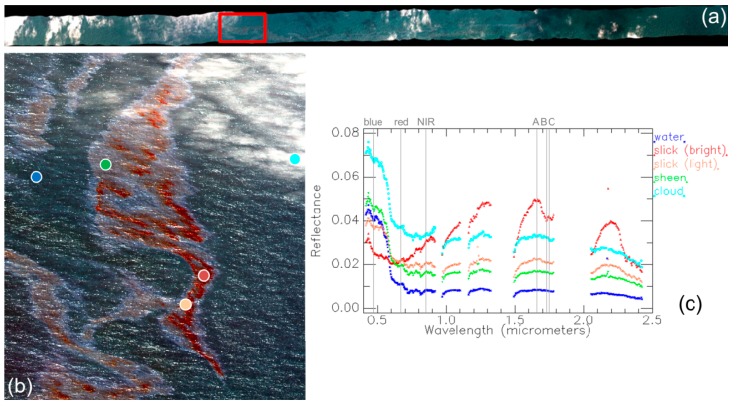
(**a**) SETHI NOFO’2015 (run 10): HS data over mineral oil on 9 June 2015 at 10:12 UTC, where red box shows the location of the ROI. (**b**) RGB composite of the ROI selected for processing. Colored circles locate the different elements of the image; and (**c**) spectral reflectance corresponding to the image elements: bright red oil slick, light red oil slick, sheen, seawater and cloud. Vertical gray lines indicate the wavelengths that are used to calculate the indices for oil detection.

Airborne data collected by SETHI on 9 June at 10:12 a.m. UTC (run 10) were selected to study the potential of hyperspectral imaging. The VNIR and SWIR images in reflectance units were registered to get access for every pixel to the full spectral signature from 0.4 to 2.5 µm. This was done using the GeFolki toolbox developed by ONERA [20] based on the common spectral range in the two images. A RGB (Red-Green-Blue) quick-look of the flight line is shown in Figure 9a. At the time of the acquisition, 10:12 a.m. UTC, there were some sparse clouds under and above the aircraft. Nevertheless, the oil slick was clearly visible overall. The red box in Figure 9a shows the location of the region of interest (ROI) that was selected for the upcoming data analysis.

A RGB color composite of the selected ROI is shown Figure 9b. This ROI was chosen for the high abundance of oil emulsion. Moreover, the presence of clouds in the image provides a robustness test of the analysis tools. Several elements are represented in the ROI: the oil slick, seawater and clouds. Within the oil slick we can distinguish three regions: a bright red region at the center of the slick, some light red pixels surrounding the first region and a whitish halo that is commonly called the sheen. The spectra acquired by the hyperspectral cameras over these five elements of the image (the three identified above within the slick as well as the clouds and seawater) are shown in Figure 9c in units of reflectance. As it can be seen, the signature of seawater (flat except for a slightly higher reflectance before 0.6 µm) is predominant for all elements except for the bright red region. This area contains the highest abundances of oil emulsion. There, oil emulsion shows a positive reflectance slope starting at 0.6 µm and three reflective peaks at 1.3, 1.65 and 2.2 µm. Furthermore, the characteristic absorption peaks of hydrocarbons are seen at 1732 and 2310 nm [21]. These spectral attributes are only visible where the emulsion abundance is high. In the case of the light red region of the oil slick, however, the emulsion abundance is less important according to the strongly faded spectral attributes of oil emulsion due to a higher presence of the seawater signature. The spectral reflectance of the sheen is very close to the signature of seawater except for a constant offset starting at 0.6 µm and a milder negative spectral slope between 0.4 and 0.6 µm. Clouds present a similar spectral signature than seawater but with a higher reflectance value.

Detection of oil slicks based on hyperspectral imaging is made possible thanks to the use of spectral indices that are based on the spectral attributes of hydrocarbons. In [22], for example, authors use a couple of spectral indices in the VNIR domain to monitor oil slicks. These are the Fluorescence Index (FI) and the Rotation-Absorption Index (RAI) that are defined as follow

(1) $$FI=\frac{\rho_{blue}-\rho_{red}}{\rho_{blue}+\rho_{red}}$$

(2) $$RAI=\left\|\rho \right\|\frac{\rho_{blue}-\rho_{NIR}}{\rho_{blue}+\rho_{NIR}}$$

where $\rho_{blue}$, $\rho_{red}$ and $\rho_{NIR}$ correspond to reflectance taken respectively at 470, 670 and 850 nm, respectively (see Figure 9c), and $\left\|\rho \right\|=\sqrt{\rho_{blue}^{2}+\rho_{NIR}^{2}}$.

**Figure 10:**
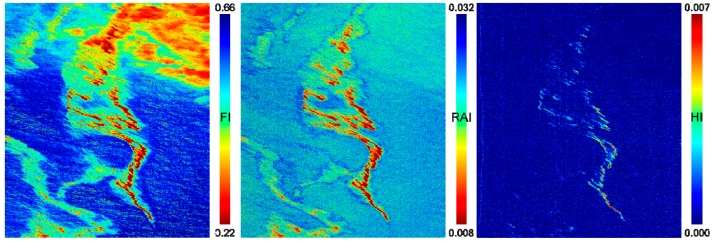
Score maps obtained with the spectral indices: (**left**) FI; (**center**) RAI; and (**right**) HI. Color code for HI is inverted in order to assign hot colors to the presence of oil emulsion.

The FI index provides information on the contrast between the blue and the red wavelengths, which is mainly shaped by the reflectance decrease of seawater before 0.6 µm. The presence of oil may reduce the spectral slope in this spectral domain. Figure 10 (left) shows the FI score map obtained from the investigated hyperspectral data. The oil slick is clearly detected, as well as its different regions (from dark red color at the center of slick to green color for the sheen). However, the high reflectance of clouds generates false alarms with FI values close to those of the slick.

The RAI index also exploits the spectral slope in the VNIR but goes further into the NIR domain by using the spectral band at 850 nm. At this wavelength, the oil emulsion reflectance may be higher than at 470 nm due to the reflectance increase of oil in the VNIR. The multiplication by the norm of the two spectral bands (2) roughly compensates the denominator and thus amplifies the difference effect (see numerator). Figure 10 (center) shows the RAI score map for the selected hyperspectral image. The slick is well detected and the clouds are less visible. However, the use of the NIR band makes the result noisy, as the VNIR camera is less performant in terms of SNR for wavelengths close to 1 micron.

Spectral indices are also available in the SWIR range for monitoring oil slicks. Among them, we selected the Hydrocarbon Index (HI), which measures the depth of the typical absorption band of hydrocarbons at 1.73 µm [23]. Although other indices are based upon the absorption peak at 2.3 µm, they are usually less performant due to the weaker absorption compared to the 1.7 µm one and the lower signal reaching the detector in this spectral range (the solar spectral illumination decreases according Planck’s law). The HI index is calculated as follows

(3) $$HI=\frac{\left(\lambda_{B}-\lambda_{A}\right)}{\left(\lambda_{C}-\lambda_{A}\right)}\left(\rho_{C}-\rho_{A}\right)+\rho_{A}-\rho_{B}$$

where $\rho_{A}$, $\rho_{B}$ and $\rho_{C}$ correspond to reflectance taken, respectively, at *λ_A_* = 1.67, *λ_B_* = 1.72 and *λ_C_* = 1.75 µm (see Figure 9c).

Figure 10 (right) shows the results of computing the HI index on the investigated hyperspectral image. The HI score map reveals the center of the oil slick, which corresponds to the bright red region in Figure 9b. However, the light red region and the sheen are not detected, as the absorption peak at 1.7 µm is visible only for high abundances of oil emulsion.

In response to the limitations observed in the previous results, we propose a new spectral index that results from the combination of the FI and RAI: the norm FI (or *nFI*). The expression for *nFI* is:

(4) $$nFI=\left\|\rho \right\|\frac{\rho_{blue}-\rho_{red}}{\rho_{blue}+\rho_{red}}$$

where $\left\|\rho \right\|=\sqrt{\rho_{blue}^{2}+\rho_{red}^{2}}$.

**Figure 11:**
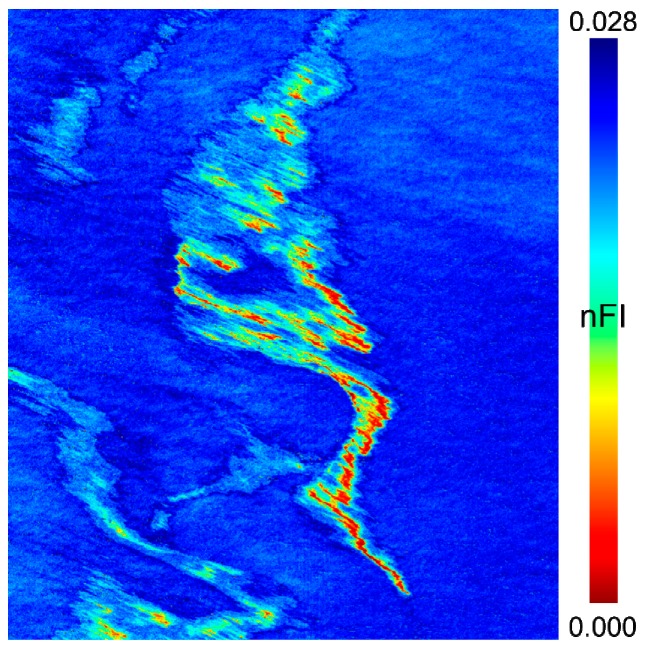
Score maps resulting from spectral index *nFI*.

Similar to FI, the index *nFI* is sensitive to the spectral slope between the blue and red wavelengths. Furthermore, *nFI* is robust against clouds due to the multiplication by the spectral norm without being affected by instrumental noise. The score map obtained with *nFI* (Figure 11) distinguishes clearly the different components of the oil slick from the background. Only little confusion exists between clouds and the fainter part of the sheen.

**Figure 12:**
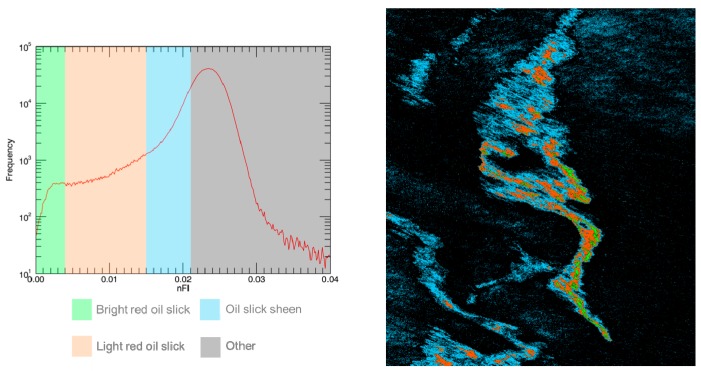
(**Left**) Histogram of the *nFI* score map with the four colored groups according to different *nFI* value ranges. (**Right**) Image in which each pixel is affected to the color corresponding to its *nFI* score group.

The results provided by the *nFI* index can be exploited to generate quick look products to be used for oil monitoring. Figure 12 (left) shows the histogram of the *nFI* score map with the peak at 0.024 corresponding to seawater. The definition of four contiguous intervals allows us to generate the image in Figure 12 (right) in which a different color has been assigned for the three regions of the oil slick (bright red, light red and sheen) and the rest of the image (seawater and clouds).

In addition to detection, hyperspectral imaging can also be used to characterize oil slicks by estimating their properties [19]. This can be achieved if the spectral properties of the oil emulsion are known in advance. Blind characterization of oil slicks (with no a priori information) is very challenging due to the variability of the spectral signature according to many parameters. One of these parameters is the age of the emulsion or the elapsed time between the generation of the emulsion and imaging of the released product.

**Figure 13:**
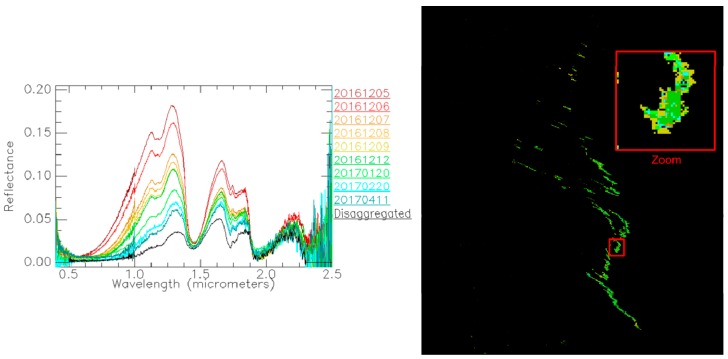
(**Left**) Spectral library of the spectral reflectance between 0.4 and 2.5 µm for the NOFO oil emulsion over time. The “disaggregated” spectrum corresponds to the measurement that was done before reconstructing the emulsion with the mixer. (**Right**) Classification map obtained with the SID approach and the spectral library. The color of pixels follows the color code of the spectral plots.

A sample of the oil emulsion was collected on the day of the campaign with the aim of characterizing the oil slick. The sample was sent by NOFO to ONERA a month later and the spectral reflectance of the emulsion, which was already in a disaggregated state, was measured later with a spectroradiometer. Afterwards, the emulsion was reconstructed on 5 December by mixing it until a homogeneous dense mixture was obtained. Spectral reflectance was regularly measured from that day on, and during several months, to study the temporal evolution of the emulsion signature. Figure 13 (left) shows the spectral library that was built using some of the measurements.

This spectral library is used in the following experiment to estimate the age of the emulsion observed by the HySpex cameras, onboard SETHI during the NOFO experiment. This is done by carrying out an image classification following a spectral matching approach. The spectral distance Spectral Information Divergence (SID) is used to compute a similarity score between a given spectrum of the investigated hyperspectral image and each class spectrum of the library [24]. Hence, each image spectrum is classified as the class providing the lowest SID score (i.e., the highest similarity). The SID approach is widely used in processing of hyperspectral images due to its high sensitivity to the shape of spectra.

Given two spectra, *x* and *y* with *N* values (*N* being the number of spectral bands), the SID score is calculated as follows

(5) $$SID\left(x,y\right)=\sum_{i=1}^{N}x_{i}^{'}\log\frac{x_{i}^{'}}{y_{i}^{'}}+\sum_{i=1}^{N}y_{i}^{'}\log\frac{y_{i}^{'}}{x_{i}^{'}}$$

where

(6) $$x_{i}^{'}=\frac{x_{i}^{}}{\sum_{i=1}^{N}x_{i}^{}},\;y_{i}^{'}=\frac{y_{i}^{}}{\sum_{i=1}^{N}y_{i}^{}}$$

For this experiment, a classification threshold was used to classify only the spectra with a SID score inferior or equal to 0.05. In this way, only the regions of the image that are similar to at least one class of the library are classified. As can be seen in Figure 13 (right), the classification map only reveals the center of the slick, which contains the purest pixels of the oil emulsion. The predominating class is the one corresponding to 12 December, thus estimating the emulsion age to be around one week. This corresponds approximately to the time lag between the preparation of the emulsion by NOFO and its release into the sea the day of the airborne campaign.

**Figure 14:**
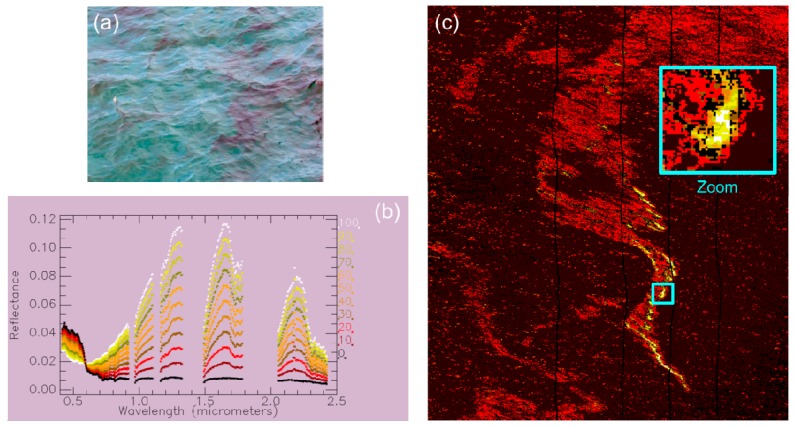
(**a**) Picture of the oil emulsion taken from the boat (note the coexistence of seawater and oil emulsion areas); (**b**) spectral library according to areal fraction in percentage; and (**c**) classification map of areal fraction. The color of pixels follows the color code of the spectral plots.

The last experiment aims at quantifying the areas of the image in which seawater coexists with the oil emulsion in the same pixel. Figure 14a shows a picture taken from the boat showing this coexistence. This phenomenon leads to observe some spectra measured by the HySpex cameras (corresponding to a 1 m^2^ region at the sea surface in the VNIR case) resulting from linear mixture of the signatures of seawater and oil emulsion. The spatial abundance of each component can be quantified by the oil areal fraction, that is, the cover fraction of the oil emulsion in a given pixel. This parameter is quantified for the investigated hyperspectral image by creating a new spectral library. Starting from a spectrum of clean seawater and a pure spectrum of the oil emulsion, several linear combinations of the two spectra are done with a step of 10%. Both endmember spectra result from averaging the spectra of a given region of the image. For the oil emulsion, the region corresponding to the bright red slick in Figure 12 is considered. The resulting spectral library is shown in Figure 14b.

The spectral matching approach SID is applied again onto the investigated hyperspectral image using the spectral library according to oil areal fraction. The resulting classification map shown in Figure 14c confirms the center of the slick as the richest region in terms of oil emulsion with an areal fraction of 70% or higher. The areal fraction drops drastically if one moves away from the center of the slick, down to 20–30% of areal fraction for the sheen. Some pixels are not classified as they do not reach the classification threshold (see black pixels). This result may point the fact that all spectral variations within the oil slick in the test image cannot be explained by the changes in areal fraction.

### 4.2. Imagery in Microwave Domain

Remote sensing techniques operating in the microwave frequencies are commonly used in offshore domain for oil slick monitoring [1,2]: firstly because the electromagnetic (EM) wave is sensitive to the modification of the sea surface induced by oil and then because radar sensors can be used any time and in almost any weather conditions. When oil is released on the ocean surface, the film layer on the top of the sea surface damps the capillary and gravity-capillary waves that are the main source of the sea surface roughness [25,26,27]. Consequently, slick-covered areas appear as dark patches in the SAR images, which make the presence of oil on the ocean surface potentially easily detectable by microwave sensors. Nevertheless, radar images must be obtained under low-to-moderate wind conditions (e.g., 2–12 m·s^−1^ at C-band [7]) to ensure enough contrast between covered and slick-free areas (lower limit) and to avoid too fast mixing of the oil into the water column (upper limit). Despite this strong interest in the use of airborne and spaceborne SAR data for oil slick detection, some issues remain unresolved when using only radar images, namely the characterization of the detected substance and the quantification of the amount of product released on the sea surface.

**Figure 15:**
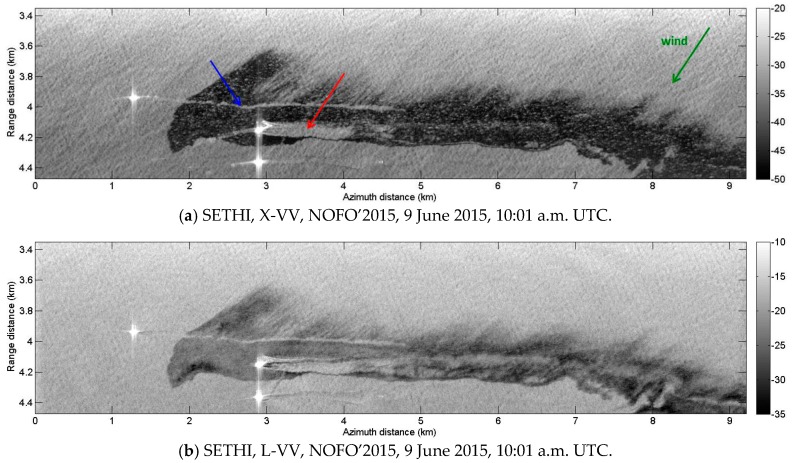
SETHI NOFO’2015 (run 8)—SAR data over mineral oil on 9 June 2015 at 10:01 a.m. UTC: (**a**) X-band VV-pol and (**b**) L-band VV-pol (multi-look 7 × 7—scale in dB).

Motivated by the need to improve the characterization of slick-covered areas from radar remote sensing, dual-frequency imagery acquired during the NOFO experiment by SETHI over a controlled release of mineral oil is investigated in the following. The oil slick is easily observable as a dark area. The backscattered signal within the slick seems more homogenous at X-band (Figure 15a) than at L-band (Figure 15b). For these images, the wind direction is from the top right (see green arrow in Figure 15a) and images show a feathered structure along the top of the slick, due to wind effect. Mineral oil accumulates at the downwind side (dark line), while the upwind side exhibits feathered features. Within the lower part of the slick, the passage of the MOS Sweeper mechanical recovery boom appears to leave behind a relatively clean sea surface (see red arrow in Figure 15a). The wake left by the passage of a ship through the slick is clearly seen (see blue arrow in Figure 15a).

Previous studies have demonstrated the effectiveness of the Polarization Difference (PD) for slick-covered area detection [28,29]. PD is defined such as

(7) $$PD=\sigma_{VV}^{0}-\sigma_{HH}^{0}$$

where $\sigma_{PP}^{0}$ is the Normalized Radar Cross Section (NRCS—in linear units) and the subscript *p* denotes either *H* or *V* polarization. As the non-polarized part of the backscattered response is removed using *PD* [28], this parameter is proportional to the spectral density of the sea surface roughness taken at the Bragg wavelength [30], *k_B_* = 2*k_EM_*sin*θ_i_*, where *k_EM_* = 2*π*/*λ_EM_* is the electromagnetic wavenumber corresponding to the radar wavelength *λ_EM_* and *θ_i_* is the local incidence angle of the *EM* wave. Hence, *PD* is mainly driven by the contribution due to short wind waves around the Bragg wavenumber, namely the capillary and gravity-capillary waves. This is precisely this scale of waves which is damped by oil when covering the ocean surface, which makes *PD* an efficient quantity for slick detection. To facilitate its use, a normalization of *PD* has been proposed in [8]

(8) $$NPD=1-\frac{PD}{PD_{seawater}}\;0\leq NPD\leq 1$$

where *PD_seawater_* is a value taken over the slick-free surface. *NPD* quantity is close to zero over clean seawater and goes to 1 as the concentration of oil locally increases.

**Figure 16:**
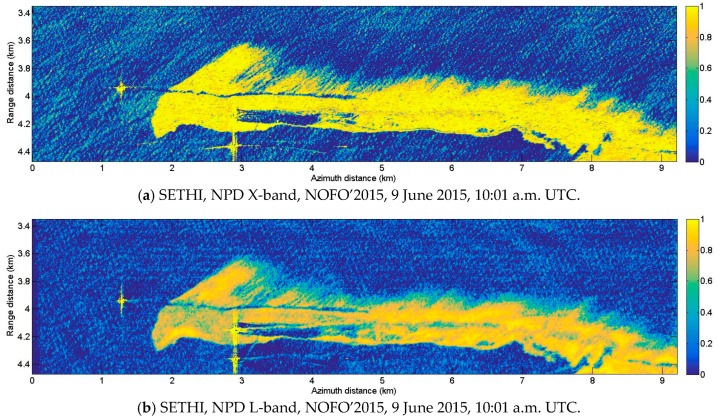
SETHI NOFO’2015 on 9 June 2015 at 10:01 a.m. UTC, NPD parameter at: (**a**) X-band and (**b**) L-band (multi-look 7 × 7).

Normalized Polarization Difference (NPD) maps computed at X- and L-band over controlled release of mineral oil are shown Figure 16. In both cases, the oil slick is easily detectable. At X-band, the slick seems homogeneous and NPD values are always close to 1 (Figure 16a), suggesting a strong damping of waves corresponding to the X-band Bragg wavelength everywhere within the spill. The NPD map at L-band (Figure 16b) looks more heterogeneous and reveals a non-uniform damping of L-band Bragg-wavelength gravity-capillary waves. These fluctuations are related to the relative concentration of oil within the slick.

**Figure 17:**
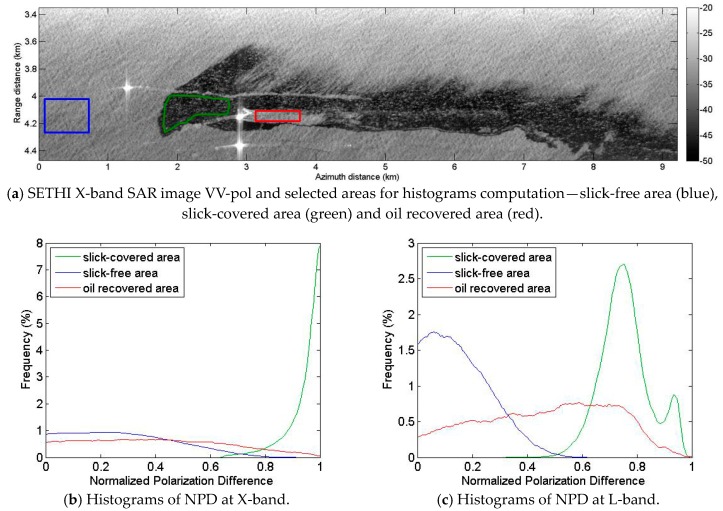
SETHI NOFO’2015 on 9 June 2015 at 10:01 a.m. UTC: (**a**) X-band VV-pol SAR image and selected areas; and histograms of NPD parameter at: (**b**) X-band; and (**c**) L-band over slick-covered (green), slick-free (blue) and oil recovered areas (red).

Histograms of NPD parameters computed at X- and L-band over clean sea, oil slick and the area behind the passage of the MOS Sweeper recovery boom are given Figure 17. Separation between histograms over contaminated (green curves) and uncontaminated areas (blue curves) confirms the robustness of the Polarization Difference for slick-sea discrimination, at both frequencies. Nevertheless, NPD values over the slick-covered area are uniformly high (close to 1) at X-band (Figure 17b—green curve) whereas at L-band (Figure 17c—green curve) one can observe a strong variation of NPD values within the oil-covered area. This supports the use of high frequencies compared to low frequencies for maritime pollution detection algorithms [25,27,31]. Histograms over the oil recovered area (Figure 17b,c—red curves) show NPD with intermediate values between those measured over slick-covered (Figure 17b,c—green curves) and slick-free areas (Figure 17b,c—blue curves), suggesting an effective surface cleaning, but also a decrease of the surface roughness comparing to that observed over clean seawater. This decrease can be explained either by the presence of hydrocarbons (in lower quantity) behind the recovery system, or by a smoothing of the sea surface caused by the MOS Sweeper boom itself.

To overcome this ambiguity, one must refer to the Polarization Ratio, defined in linear units as

(9) $$PR=\frac{\sigma_{HH}^{0}}{\sigma_{VV}^{0}}$$

**Figure 18:**
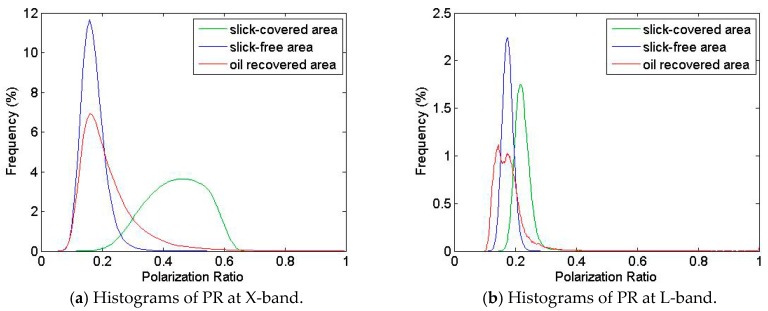
SETHI NOFO’2015 on 9 June 2015 at 10:01 a.m. UTC, histograms of PR parameter at: (**a**) X-band; and (**b**) L-band over slick-covered (green), slick-free (blue) and oil recovered areas (red).

The Polarization Ratio (PR) depends on the local incidence angle and the relative dielectric constant but is, to the first order, independent of the sea surface roughness [32]. Thus, PR allows distinguishing between slick-covered sea surface and oceanographic phenomena [29] through the difference in the dielectric constant between the clean sea surface and the polluted seawater [32]. At L-band, the thickness of the oil-slick is probably too low (typically, the thickness of such layer is of the order of micrometer to millimeter [33]) compared to the penetration depth to be seen by the radar and to affect the Polarization Ratio (Figure 18b). However, at X-band (with typical value of penetration depth less than millimeter), histograms over contaminated (Figure 18a—green curve) and uncontaminated (Figure 18a—blue curve) areas are sufficiently apart to be separated. The histogram of PR values obtained over the oil-recovered area (Figure 18a—red curve) shows a “heavy tailed” distribution with a mean value close to that obtained over the slick-free area. This suggests that, overall, the sea has been reasonably well cleaned despite some areas that remain locally covered by oil.

## 5. Summary of Performances Assessment of Multi-Wavelength Imagery for Oil-Slick Monitoring

In the optical domain, VNIR and SWIR hyperspectral cameras have been operated with a very high spectral resolution. Based on imagery collected by SETHI over mineral oil-slick, key results have been given. First, a new spectral index in the VNIR domain, called nFI (norm Fluorescence Index), is proposed. It is efficient to detect oil-covered sea surface, while being robust against clouds without suffering from low instrumental SNR. In addition to detection, estimation of oil-covered surface properties by hyperspectral sensors has been explored. The employed methodology is based on the Spectral Information Divergence (SID) which computes a measure of similarity between the spectral response (pixel-based approach) collected by SETHI and each class spectrum of a given library. The originalities proposed in this paper are: first, the operated sensors acquire the full spectral range of 410–2500 nm with a very high spectral resolution. Secondly, the spectrum library used has been specifically designed for this purpose. It includes spectral reflectance regularly measured in laboratory for several months, which allows the study of the temporal evolution of the oil spectral signature. Following this approach, the estimated age of the hydrocarbons imaged by hyperspectral sensors onboard SETHI is in agreement with the time lag between the preparation of the emulsion by NOFO and its release into the sea the day of the exercise. Quantifying the areas of the image in which seawater coexists with the oil emulsion in the same pixel has also been assessed. The results show that the oil areal fraction has a strong impact on the spectral signature. As expected, the center of the slick is the part with the highest spatial abundance of oil emulsion. However, the obtained classification map shows that all spectral variations within the oil slick cannot be explained only by the changes in areal fraction.

In the microwave domain, studies based on data acquired by the SIR-C/X-SAR imaging radar system flown aboard the space shuttle in 1994 and operating at L- (1.275 GHz), C- (5.172 GHz) and X-bands (9.677 GHz) have assessed performances of multi-frequency SAR imagery for maritime pollution monitoring. As an example, it has been demonstrated in [27] the capability of such data to distinguish between mineral oil and biogenic films (look-alike). As no multi-frequency instrument has been launched in space since this mission, researchers have had little opportunities to pursue multi-frequency analyses over slick-covered areas. Studies using SAR data acquired in several frequency bands over sea surface covered by oil are proposed in [11,34], but multi-frequency images are not simultaneous and are acquired by spaceborne sensors characterized by a high instrumental noise floor. X- and L-band polarimetric SAR data acquired by SETHI during the oil-on-water exercise and related analysis presented in this paper demonstrate the added value brought by multi-frequency polarimetric radar sensors to monitor sea surface covered by mineral oil. Using different EM wavelengths, the radar signal simultaneously interacts with hydrodynamic mechanisms of different scales (related to the Bragg wavelength) and then increased information collected by the remote sensing system. High frequency (e.g., X-band) is suitable for detecting oil-covered areas [25] and also for assessing the efficiency of a mechanical recovering system operating during a decontamination phase. Lower frequency imagery (e.g., L-band) permits to evaluate the relative concentration of oil within a slick-covered area and then guide response activities towards the areas that are the most affected by the released substance. The efficiency of results obtained at X- and L-band and presented herein relies in the very low instrumental noise floor offered by SETHI, which allows valid analysis of oil-covered sea surface properties.

## 6. Conclusions

SETHI, the airborne sensor developed by ONERA, is a unique system of imagery designed to explore the science applications of remote sensing. It allows the quasi-simultaneous acquisition of high spatial resolution data in both optical and microwave domains. Up to three different Radar bands can be operated at the same time and each sensor is characterized by a very low instrumental noise floor, not currently available from space. Hyperspectral cameras cover the full range of 0.4–2.5 µm with a very high spectral resolution and one panchromatic camera collects data aligned with the radar observation.

Hyperspectral and radar sensors embedded onboard SETHI have been operated during the 2015 oil-on-water exercise where controlled releases of mineral oil have been carried out by NOFO in the North Sea, Europe. The acquired dataset is therefore unique because of the overall high resolution, the low noise levels and the quasi-simultaneous acquisitions at L-band, X-band and over the full hyperspectral range. In this paper, dual-frequency SAR data and hyperspectral images have been shown to provide information on the quantity and the nature of oil discharged in oceans. It has also been reported how multi-wavelength remote sensing data can assess the efficiency of the cleanup operations. The work on this dataset has just began with a separate analysis of the potentials of both radar and hyperspectral imagery. They provide different information: the relative concentration and the nature of the oil. However, because the acquisitions are almost simultaneous, the dataset provides a unique opportunity to explore the synergy between the two types of imagery. One first observation is that the extent of the observed slick is much larger with radar than with optical imagery.

This complementary has not yet been addressed and will be studied in future work, both with the joint analysis of the images and with associated ocean surface modeling.

## Figures and Tables

**Table 1:** Environmental Conditions during the MOS Sweeper experiment.

Date	Time (UTC)	Wind Speed at 10 m (m·s^−1^)	Wind Direction (from-deg)	Significant Wave Height (m)
9 June 2015	06:00	5	250	1
	09:00	5	250	1
	12:00	7	250	1

**Table 2:** Main parameters of optical sensors operated during the NOFO oil-on-water exercise.

Optical Sensors	HySpex VNIR1600	HySpex SWIR320m-e	Panchromatic CamV2
Number of pixels	1600	320	7256 × 5462
Field of view	17°	14°	27°
Spectral domain	0.4–1.0 µm	1.0–2.5 µm	0.4–0.8 µm
Spectral sampling	3.6 nm	6 nm	-
Ground sampling distance (across and along track) at 9000 ft	0.51 × 1.03 m	2.06 × 2.06 m	0.17 × 0.17 m

**Table 3:** Radar waveform operated during the NOFO oil-on-water exercise.

Radar Sensors	SAR X-Band	SAR L-Band
Carrier frequency	9.75 GHz	1.325 GHz
Bandwidth/Slant range resolution	300 MHz/0.5 m	150 MHz/1 m
Pulse Repetition Frequency (PRF)	5000 Hz	5000 Hz
Pulse duration	20 µs	20 µs
Sampling frequency	800 MHz	800 MHz

**Table 4:** Remote sensing data acquired by SETHI during the MOS Sweeper trial (9 June 2015). Data investigated herein appear in bold letters.

Run	Time (UTC)	Sensor	Flight Heading (deg)	Viewing Direction (deg)
1	09:32	HS VNIR + SWIR	288°	Nadir
2	09:34	HS VNIR + SWIR	290°	Nadir
3	09:37	PolSAR X + L	108°	198°
4	09:43	HS VNIR + SWIR	292°	Nadir
5	09:46	PolSAR X + L	113°	203°
6	09:52	HS VNIR + SWIR	294°	Nadir
7	09:55	PolSAR X + L	113°	203
**8**	**10:01**	**PolSAR X + L**	**293°**	**023°**
9	10:08	HS VNIR + SWIR	298°	Nadir
**10**	**10:12**	**HS VNIR + SWIR**	**116°**	**Nadir**
11	10:17	HS VNIR + SWIR	293°	Nadir
12	10:20	PolSAR X + L	108°	198°
13	10:24	HS VNIR + SWIR	286°	Nadir
14	10:27	PolSAR X + L	107°	197°
